# *TP53* p.R337H prevalence in a series of Brazilian hereditary breast cancer families

**DOI:** 10.1186/1897-4287-12-8

**Published:** 2014-03-13

**Authors:** Nathalia M Cury, Victor EF Ferraz, Wilson A Silva

**Affiliations:** 1 Department of Genetics, Ribeirão Preto Medical School, University of São Paulo (FMRP/USP), São Paulo, Brazil; 2 National Institute of Science and Technology in Stem Cell and Cell Therapy (INCTC), Center for Cell Therapy (CTC) and Regional Blood Center of Ribeirão Preto, Ribeirão Preto, Brazil; 3 Center for Medical Genomics, HC-FMRP/USP, Ribeirão Preto, Brazil

**Keywords:** Breast cancer, *TP53* mutation, *BRCA1*, High resolution melting

## Abstract

**Background:**

Approximately 5-10% of breast cancers are hereditary. Among hereditary syndromes, Hereditary Breast and Ovarian Cancer Syndrome (HBOC) and Li-Fraumeni Syndrome (LFS) have received the most attention. HBOC is due to mutations in the *BRCA1* and *BRCA2* genes and is characterized by breast adenocarcinoma and/or epithelial ovarian carcinoma. LFS is associated with germline mutations in *TP53*; the most frequent cancer types associated with this syndrome are sarcoma, breast cancer, leukemia, brain tumors and adrenocortical carcinomas. Other cancers related to LFS are found at lower frequencies. In Brazil, especially in the southern part of the country, a specific mutation in the *TP53* gene, *TP53* p.R337H, occurs at a high frequency in childhood adrenocortical tumors. It has been proposed that this mutation increases breast cancer risk in southern Brazilian women.

**Methods:**

We carried out a case-control study to determine the prevalence of the *TP53* p.R337H mutation in 28 female cancer patients attended at the Cancer Genetic Counseling Service of the General Hospital of the University of São Paulo Medical School of Ribeirão Preto who fulfilled Hereditary Breast and Ovary Cancer Syndrome genetic test criteria compared to healthy woman (controls). *TP53* p.R337H mutation status was determined using the High Resolution Melting (HRM) method, followed by DNA sequencing. Fisher’s test was used to compare the prevalence of *TP53* p.R337H in the patient and control groups.

**Results:**

Two of the breast cancer cases (7.1%) and none of the controls carried the *TP53* p.R337H mutation. At the time of the investigation, both cases fulfilled testing criteria for Hereditary Breast and Ovary Cancer Syndrome but not Li-Fraumeni or Li-Fraumeni-like Syndrome, based on genetic testing criteria of NCCN Clinical Practice Guidelines in Oncology (v.1.2010).

**Conclusions:**

We suggest that genetic screening of Brazilian breast cancer patients who fulfill Hereditary Breast and Ovary Cancer Syndrome criteria and have a family history that includes other tumors of the LFS/LFL spectrum be tested for the *TP53* p.R337H mutation.

## Background

The World Health Organization (WHO) estimates that over one million women are affected annually by breast cancer [1]. In Brazil, breast cancer is the most prevalent cause of death due to cancer among women from 40 to 69 years old [2]. About 5-10% of breast cancers are hereditary, and approximately 30% of young women who develop this type of cancer have a predisposition to disease [3-6]. Two major hereditary cancer predisposition syndromes are related to hereditary breast cancer phenotype: Hereditary Breast and Ovarian Cancer Syndrome (HBOC) and Li-Fraumeni Syndrome (LFS). HBOC is due to mutations in the *BRCA1* and *BRCA2* genes; it is characterized by ductal or lobular breast adenocarcinoma and epithelial ovarian carcinoma [7].

In 1990, LFS was found to be associated with germline mutations in *TP53*[8]. Pathogenic mutation carriers for this gene have a cumulative risk of up to 90% for the development of a cancer spectrum, which is usually diagnosed before the age of 45. The most frequent cancer types include sarcoma, breast cancer, brain tumors, leukemia and adrenocortical carcinomas. Other cancers are observed at a lower frequency, including lymphomas, gastric cancer and melanoma [9,10]. Families that do not have the classic phenotype of this syndrome are called Li-Fraumeni like (LFL) or Li-Fraumeni variant (Table 1) [11-13]. The NCCN Clinical Practice Guidelines in Oncology v.4.2013 take into account only classic LFS and Chompret criteria for LFS/LFL genetic testing [14].

**Table 1:** Current genetic testing criteria for LFS

**Classification**	**Description**
Classic (Li et al., 1988) [25]	Proband diagnosed with sarcoma before age 45, and a FDR with cancer before age 45, and another first- or second-degree relative with any cancer diagnosed under age 45 or with sarcoma at any age
Birch (Birch et al.,1994) [11]	Proband with any childhood cancer or sarcoma, brain tumor, or adrenocortical carcinoma diagnosed under 45 years, and a FDR or SDR with a typical Li-Fraumeni syndome-related cancer (sarcoma, breast cancer, brain tumor, leukemia, or adrenocortical carcinoma) diagnosed at any age and an FDR or SDR in the same genetic lineage with any cancer diagnosed under the age of 60
Eeles (Eeles, 1995) [12]	Two different tumors that are part of the extended Li-Fraumeni syndome in FDR or SDR at any age (sarcoma, breast cancer, brain tumor, leukemia, adrenocortical tumor, melanoma, prostate cancer, and pancreatic cancer)
Revised Chompret (Tinat et al., 2009) [26]	I—Proband diagnosed with tumor belonging to Li-Fraumeni syndome spectrum (soft tissue sarcoma, osteosarcoma, brain tumor, premenopausal breast cancer, adrenocortical carcinoma, leukemia, lung bronchoalveolar cancer) before age 46 and at least one first- or second degree relative with Li-Fraumeni syndrome cancer (except breast cancer if the proband has breast cancer) under the age of 56 years or with multiple tumors at any age
	II—Proband with multiple primary tumors (except multiple breast), two of which belong to Li-Fraumeni syndome tumor spectrum and the first of which occurred before age 46
	III—Proband with adrenocortical carcinoma or choroid plexus tumor at any age, irrespective of family history

*FDR*, First-degree relative; *SDR*, Second-degree relative.

Given the similar mutation rates of the *BRCA1*, *BRCA2* and *TP53* genes, Lee *et al*. [15] proposed that women who developed breast cancer before 30 years of age should undergo genetic testing for all three genes simultaneously. Custodio et al. (2013) estimated an overall frequency of 0.2-0.3% for *TP53* p.R337H in a southern Brazil state [16]. This mutation has been identified in >90% of Brazilian patients with childhood adrenocortical carcinoma, a rare but remarkably frequent tumor in southeastern and southern Brazil [16]. It also has been proposed that this mutation increases breast cancer risk in women, especially in southern Brazil [17-19]. The frequency of *TP53* p.R337H in women with breast cancer has been reported as 2.4% [18], while in healthy women it is only 0.3% [20].

Given this potential association with breast cancer and the high frequency of *TP53* p.R337H in southern Brazil, we conducted a case-control study to compare *TP53* p.R337H mutation prevalence in healthy controls with that in female breast cancer patients in Ribeirão Preto, Sao Paulo state, located in southeast Brazil. The affected women fulfilled HBOC genetic test criteria, according to NCCN Clinical Practice Guidelines in Oncology v.1.2010 [21].

## Methods

### Subjects

This study was approved by the Ethics Committee of the University of Sao Paulo Medical School of Ribeirão Preto, SP, Brazil; informed consent was obtained from participants. We analyzed 28 DNA samples of unrelated women diagnosed with breast cancer that fulfilled the criteria for HBOC genetic testing according to NCCN Clinical Practice Guidelines in Oncology v.1.2010 [21] (Table 2). All patients were enrolled in the Cancer Genetic Counseling Service of the General Hospital of the University of São Paulo Medical School of Ribeirão Preto (HCFMRP-USP).

**Table 2:** **Patient criteria for genetic testing according to NCCN clinical practice guidelines in oncology v.1.2010 **[21]**for HBOC**

**Patient 1**	**Patient 2**	**Criteria**
	**X**	Personal history of breast cancer ≤ 45 years
		Personal history of two primary breast cancers, the first being diagnosed before 50 years of age
		Personal history of breast cancer with age at diagnosis < 50 years with limited family history
	**X**	Personal history of breast cancer ≤ 50 years with ≥ 1 relative with breast cancer ≤ 50 years and/or ≥ 1 relative with epithelial ovarian cancer, fallopian tube cancer, or primary peritoneal cancer at any age
		Personal history of triple-negative breast cancer, with age at diagnosis < 60 years
**X**		Personal history of breast cancer at any age, with ≥ 2 relatives with breast cancer and/or epithelial ovarian cancer, fallopian tube cancer, or primary peritoneal cancer at any age
		Personal history of breast cancer + Personal history of epithelial ovarian cancer, fallopian tube cancer, or primary peritoneal cancer

The control group was formed by 120 healthy women, age-matched to cases (Table 3), without family history of cancer, randomly selected among women attended at HCFMRP-USP and unrelated to the patients.

**Table 3:** Patient and control group clinical features

**Features**	**Patient group**	**Control group**
Number	28	120
Age (years)	41.75 (±10.25)	41.95 (±10.58)
Gender	F: 28 (100%)	F: 120 (100%)

*F*, Female.

### Sample collection

Peripheral blood samples (10 mL) were collected into vacutainer tubes containing EDTA, while they were attended at the clinic. Genomic DNA was extracted using the Wizard Genomic DNA Purification Kit (Promega, Madison, WI, USA), following the manufacturer’s recommendations and stored at -20°C until genotyping analysis. The samples were collected from March 2009 to June 2010.

### Genotype determination

#### High Resolution Melting (HRM)

The HRM method was used to detect the *TP53* p.R337H mutation. The HRM primer sequences were as described by Bastien *et al*. [22]. Reactions were performed with a total volume of 20 uL (3.6 uL of MiliQ water, 1.2 mL of each primer at 5 pmol/uL, 10 uL of MeltDoctor HRMTM Master Mix (Applied Biosystems) and 4 uL of DNA at 5 ng/uL). The amplification parameters were: 95°C for 10 minutes, 40 cycles of 95°C for 15 seconds and annealing temperature for 1 minute. For melting curve analysis, the parameters were: 95°C for 10 seconds, 60°C for 1 minute, 95°C for 15 seconds and 60°C for 15 seconds. The samples with different melting curves were sequenced to validate and characterize the mutation.

#### DNA sequencing

For *TP53*, the different melting curve fragments were sequenced in an automatic sequencer XL 3500 Genetic Analyzer (Applied Biosystems). The reaction consisted of 1 to 2 uL of amplified DNA, 2 uL of Big Dye Terminator v3.1 Cycle, 2 uL of 5X Sequencing Buffer (Applied Biosystems), 1 uL of primer and sufficient water to complete 10 uL. The amplification parameters of the sequencing reaction were: 95°C for 1 minute followed by 25 cycles of 95°C for 10 seconds, 50°C for 5 seconds and 60°C for 4 minutes.

The complete coding sequence and exon-intron boundaries of *BRCA1* and *BRCA2* were analyzed in two *TP53* p.R337H positive females. The sequences of the primers were those described by Leeneer et al. [23], and Keshavarzi et al. [24], respectively.

### Statistical analyses

The *TP53* p.R337H mutation frequency in breast cancer patients and controls was compared using Fisher’s exact test with GraphPad Prism 5 software to calculate odds ratios (OR), with confidence intervals (CIs) of 95%. A p-value of <0.05 was considered to be statistically significant. The *TP53* p.R337H frequency in breast cancer patients was compared to the estimated Brazilian population frequency [16], which we assumed to be the true prevalence rate for the Brazilian population, using the one-sided exact test for binomial proportions.

## Results

**Figure 1:**
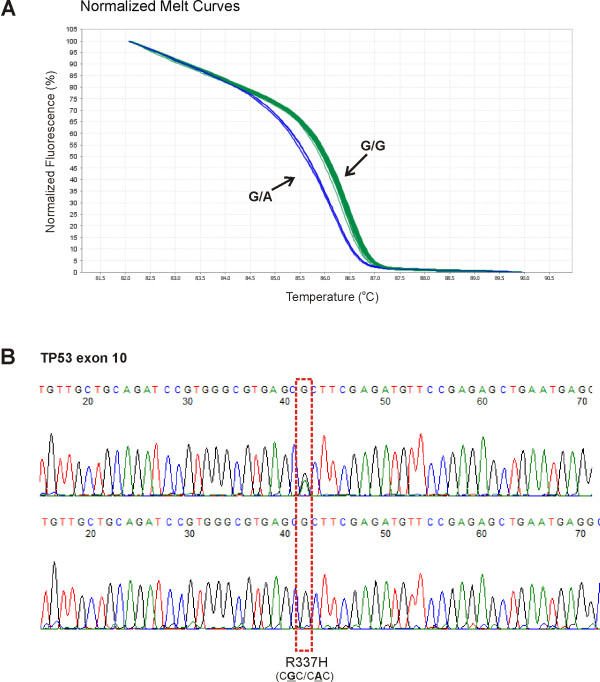
***TP53 *p.R337H mutation detection. A)** Melting curves for each specific genotype identified in exon 10 of the *TP53* gene containing the *TP53* p.R337H mutation; **B)** Sequencing results of a heterozygous *TP53* p.R337H mutation sample (melting curve in blue) and a wild type sample (melting curves in green).

Twenty-eight women diagnosed with breast cancer were tested for *TP53* mutations; two of them (7.1%) were found to carry a pathogenic mutation, heterozygous *TP53* p.R337H (Figure 1). Both are negative for *BRCA1* and *BRCA2* pathogenic mutations. *TP53* p.R337H is known to be more frequent in women with breast cancer, especially in families in southern Brazil suspected to have Li-Fraumeni Syndrome [17].

**Figure 2:**
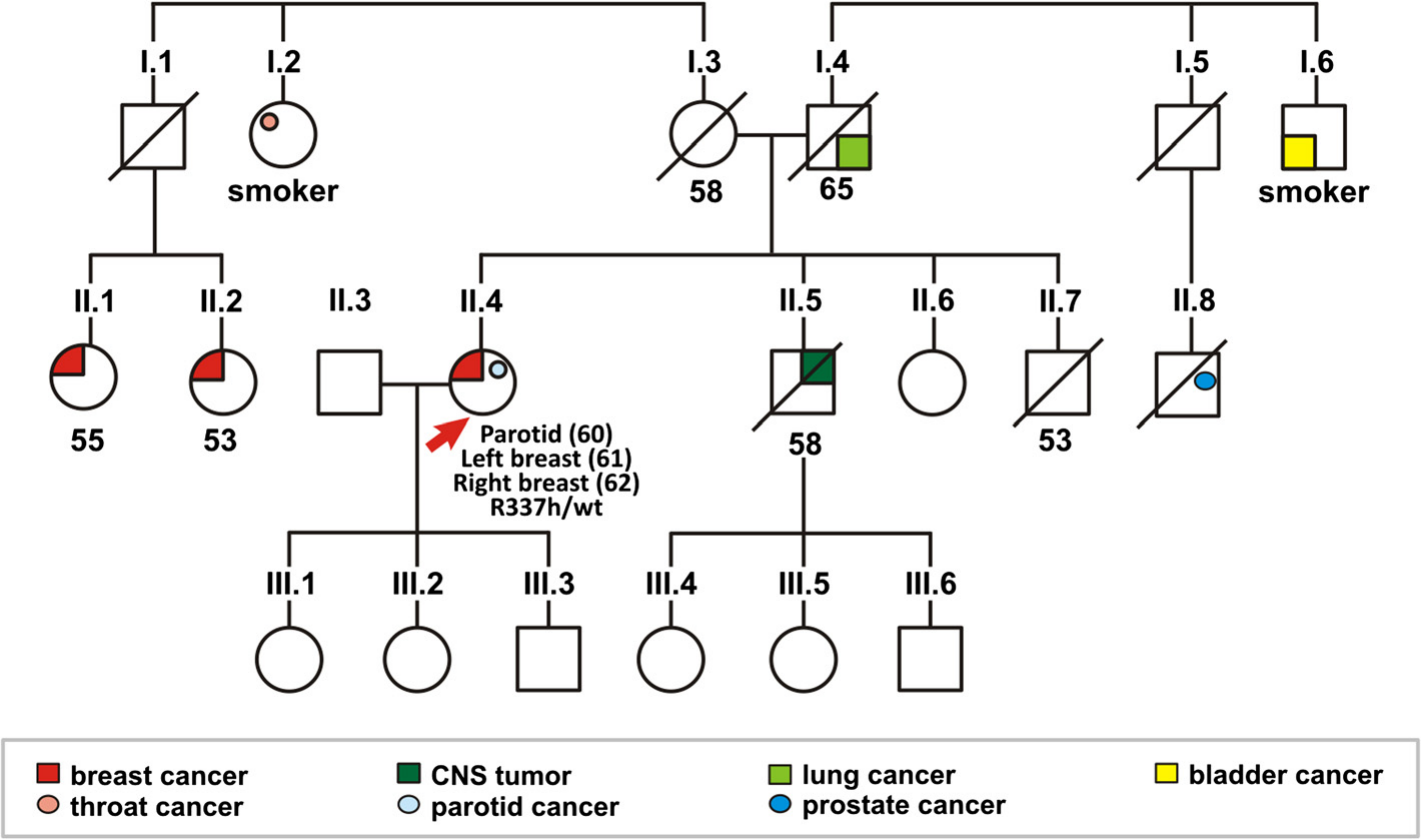
**Pedigree of patient 1, showing cancer history and the *TP53 *p.R337H mutation.** Arrow indicates the proband; present age or age at death is indicated below individuals. The cancer diagnosis age is indicated in brackets. wt = wild type.

Patient 1 was diagnosed with parotid cancer at age 60 and bilateral breast cancer at age 61 in the left breast and age 62 in the right breast. Her brother had Central Nervous System cancer at age 58. Nevertheless, she did not meet the criteria for Li-Fraumeni Syndrome according to NCCN Guidelines v.1.2010 [21] and v.4.2013 [14], because the tumors related to the Li-Fraumeni Syndrome spectrum, in this case breast and prostate cancer, were diagnosed in third degree relatives (Figure 2). In the context of HBOC, she fulfilled the criteria of bilateral breast cancer personal history at any age, as two third-degree relatives were also diagnosed with breast cancer (Table 2).

**Figure 3:**
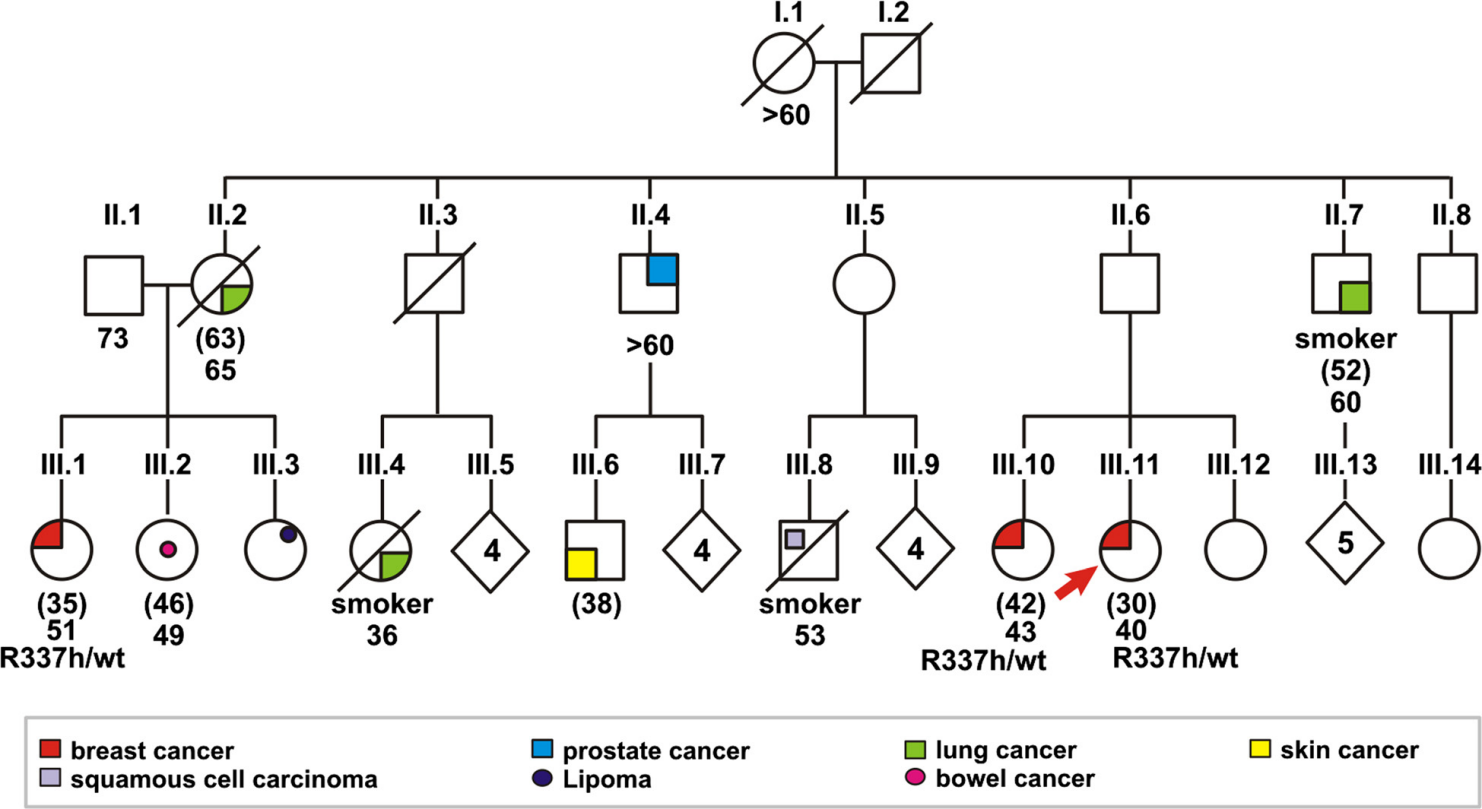
**Pedigree of patient 2, showing cancer history and the *TP53 *p.R337H mutation.** Arrow indicates the proband; present age or age at death is indicated below individuals. The cancer diagnosis age is indicated in brackets. wt = wild type.

Patient 2 was diagnosed with breast cancer at age 30. Her sister and her cousin were diagnosed with breast cancer at ages 42 and 35, respectively. Both had the same *TP53* mutation; they were heterozygous for *TP53* p.R337H. Additionally, her uncle was diagnosed with prostate cancer after he turned 60. Other tumors that are not part of the Li-Fraumeni Syndrome spectrum were diagnosed in second and third-degree relatives (Figure 3). There were two HBOC criteria for this patient: breast cancer personal history before 45 years old and family history of breast cancer before 50 years old; one first-degree relative and one third-degree relative were diagnosed with breast cancer before they were 50 (Table 2).

The HBOC criteria fulfilled for all 28 patients based on NCCN Guidelines v1.2010 [21] continued without change in v4.2013 [14].

Though both patients had a family history that included other tumors at the time of investigation, they did not fulfill the criteria for Li-Fraumeni Syndrome, according to NCCN Clinical Practice Guidelines in Oncology v.1.2010 [21]. However, the v.4.2013 [14] of these guidelines (Table 4) indicated that individuals with breast cancer by 35 years of age, not before 30 years of age as in previous version, and negative for *BRCA1*/2 fulfills LFS testing criteria. Consequently, according to the latest NCCN guidelines, patient 2 fulfills the criteria for LFS. In addition to the classic LFS [25] and Chompret [26] criteria, there are two more clinical criteria, called Birch [11] and Eeles [12]. Patient 1 just meets Eeles criteria for LFL, the less stringent one.

**Table 4:** **Genetic testing criteria according to NCCN clinical practice guidelines in oncology v.4.2013 **[14]**for LFS**

**Criteria**	**Description**
**Familial TP53 mutation**	Individual from a family with a known TP53 mutation
**Early-onset breast cancer**	Personal history of breast cancer ≤ 35 years of age with a negative BRCA1/2 test
**Classic LFS criteria**	Personal history of sarcoma < 45 years + 1 first-degree relative with cancer diagnosed at age <45 years + 1 first- or second-degree relative in the same lineage with cancer diagnosed at age < 45 years or sarcoma at any age
**Chompret criteria**	Personal history of a tumor of the LFS spectrum (eg. soft tissue sarcoma, osteosarcoma, brain tumor, breast cancer, adrenocortical carcinoma, leukemia, lung bronchoalveolar cancer) before 46 years of age + at least one first- or second-degree relative with any of the aforementioned cancers (other than breast cancer if the proband has breast cancer) before the age of 56 years or with multiple primaries at any age
	Personal history of multiple tumors (except multiple breast tumors), two of which belong to the LFS tumor spectrum, with the initial cancer occurring before the age of 46 years
	Personal history of adrenocortical carcinoma or choroid plexus carcinoma at any age of onset, regardless of family history

*LFS*, Li-Fraumeni Syndrome.

In comparison with the two of 28 HBOC breast cancer women patients who had the *TP53* p.R337H mutation, none of the 120 healthy women without family history of cancer had this mutation. We found a significant association of *TP53* p.R337H mutation with breast cancer (p = 0.0347; Table 5). Comparing our results with the overall frequency of *TP53* p.R337H in southern Brazil, assumed by Custodio et al. (2013) to be in the range of 0.2–0.3% [16], we found that the *TP53* p.R337H mutation frequency was significantly higher among breast cancer women with HBOC than in the general Brazilian population (p = 0.001 and 0.003 in comparison to 0.2 and 0.3%, respectively; one-sided exact binomial test).

**Table 5:** TP53 P.R337H frequency in breast cancer patients with HBOC criteria and control groups

**TP53 P.R337H**	**Control (n = 120)**	**Breast cancer (n = 28)**	**P**	**OR 95% CI**
Arg/Arg	120 (100%)	26 (92.9%)	0.0347	0.0439 (0.0020 to 0.9438)
Arg/His	0	2 (7.1%)		

## Discussion

Based on our study, we suggest that *TP53* p.R337H mutation prevalence in breast cancer patients suspected of HBOC in Ribeirão Preto, São Paulo, Brazil is high (7.1%). This result falls within the range of that found in previous studies conducted in southern Brazil (2.4-13%) [17,18]. However, the study by Achatz et al. is based on LFS/LFL families with tumors other than breast cancer.

This mutation is responsible for exchanging an arginine for a histidine (CGC to CAC) at codon 337, located at an oligomerization domain of p53 protein. It has been primarily associated with adrenocortical tumors in children [27,28].

Contact of alpha-helices of two adjacent monomers through a hydrogen bond is critical to oligomer stability and hence for p53 binding to DNA. Because histidine pKa is lower than arginine pKa, increased pH conditions (pH 8.3) cause histidine deprotonation, making it incapable of forming a hydrogen bond, consequently preventing protein binding to DNA [27,29]. However, the pathogenicity of *TP53* p.R337H is still questionable due to the low number of functional studies.

Achatz *et al*. (2007) [17] screened for the *TP53* p.R337H mutation in 45 Brazilian subjects from unrelated families with cancer histories suggestive of LFS. They found the mutation in six cases (13%). Interestingly, the most common tumor type in these families was breast cancer (30.4%).

Assumpção et al. (2008) [18] estimated the prevalence of this mutation in 123 breast cancer cases and 223 age-matched controls. Three of the cases (2.4%) and none of the controls were carriers of the *TP53* p.R337H mutation (P = 0.04). All three cases were relatively young at diagnosis (range 19-44 years old), and two of the three cases had a family history suggestive of LFS.

The role of the *TP53* p.R337H mutation in breast carcinogenesis is still unclear. Achatz et al. (2007) found that, in an invasive ductal breast adenocarcinoma sample, the *TP53* p.R337H mutation was homozygous in tumor tissue and heterozygous in peripheral blood, suggesting a role in tumor development. However, Assumpção et al. (2008) found the mutant *TP53* p.R337H allele to be absent in the three breast tumors examined.

Gomes *et al*. (2012) [30] evaluated *TP53* p.R337H mutation frequency in 390 unselected breast cases and 324 controls from Rio de Janeiro state. Two of the breast cancer cases (0.5%) and none of the controls carried the mutation. Both cases had an early age at diagnosis (<40 years old) and a family history including breast and other cancer types.

Our study shows that *TP53* p.R337H is found in women who fulfill HBOC genetic testing criteria but not the LFS criteria, according to NCCN Clinical Practice Guidelines in Oncology v.1.2010 [21]. The change made in v.4.2013 [14] of these guidelines led us to include patient 2 in LFS genetic testing criteria, but not patient 1. This patient potentially fulfills the Eeles criteria, which is not included in NCCN testing criteria. This fact, associated with the apparently high frequency of *TP53* p.R337H mutation in the population that we analyzed, reinforces the necessity of adjusting genetic testing criteria for hereditary syndromes according to local populations characteristics.

It has been suggested that *TP53* genetic testing should be considered for women diagnosed with breast cancer under age 30 after they have previously tested negative for mutations in *BRCA1* and *BRCA2*[31]. However, given similar mutation rates in early breast cancer, Lee *et al*. (2012) [15] proposed that these women should undergo genetic testing for mutations in all three genes at the same time. Taking into account the high frequency of the *TP53* p.R337H mutation in Brazilian women with breast cancer [17,18,20,30], and given that the *TP53* p.R337H genetic test (single nucleotide change at codon 337) is easy, fast and inexpensive, we suggest that the *TP53* p.R337H mutation screening should not be restricted to early breast cancer patients, but to all Brazilian breast cancer patients with a family history that includes other LFS/LFL tumors.

One important limitation of our study is the relatively small number of subjects. Nevertheless, the association of *TP53* p.R337H mutation with breast cancer that we found is enough to lead us to suggest that *TP53* p.R337H mutation screening should be conducted at the same time as BRCA testing and not necessarily only after patients have previously tested negative for BRCA gene mutations. This could speed up diagnosis of breast cancer and help optimize genetic counseling procedures.

## Conclusions

Our study shows that *TP53* p.R337H mutation prevalence in breast cancer patients suspected of Hereditary Breast and Ovary Cancer Syndrome in Ribeirão Preto, São Paulo, Brazil is high (7.1%) and could play an important role in predisposition to breast cancer in this population. However, there is currently no robust evidence for a role of this mutation in breast carcinogenesis.

We propose that *TP53* p.R337H mutation screening be conducted in Brazilian women who have been diagnosed with breast cancer at any age, who fulfill Hereditary Breast and Ovary Cancer Syndrome genetic testing criteria and have a family history that includes other tumors of the LFS/LFL spectrum.

## Abbreviations

BRCA1: Breast cancer susceptibility gene 1; BRCA2: Breast cancer susceptibility gene 2; HBOC: Hereditary breast and ovary cancer syndrome; HCFMRP-USP: General Hospital of the University of São Paulo Medical School of Ribeirão Preto; HRM: High resolution melting; CIs: Confidence intervals; LFL: Li-Fraumeni like; LFS: Li-Fraumeni syndrome; NCCN: National Comprehensive Cancer Network; OR: Odds ratio; PCR: Polymerase chain reaction; WHO: World Health Organization.

## Competing interests

The authors declare that they have no competing interest.

## Authors’ contributions

WASJ conceived the study. NMC participated in the study design, conducted the mutation analysis, statistical analysis and manuscript preparation. VEFF participated in the study design, acquisition of data, statistical analysis and revised the manuscript critically. All authors read and approved the final manuscript.
